# Utility of targeted next generation sequencing for inborn errors of immunity at a tertiary care centre in North India

**DOI:** 10.1038/s41598-022-14522-1

**Published:** 2022-06-21

**Authors:** Amit Rawat, Madhubala Sharma, Pandiarajan Vignesh, Ankur Kumar Jindal, Deepti Suri, Jhumki Das, Vibhu Joshi, Rahul Tyagi, Jyoti Sharma, Gurjit Kaur, Yu-Lung Lau, Kohsuke Imai, Shigeaki Nonoyama, Michael Lenardo, Surjit Singh

**Affiliations:** 1 Allergy Immunology Unit, Department of Pediatrics, Advanced Pediatrics Centre, Post Graduate Institute of Medical Education and Research, Chandigarh, 160012 India; 2 Department of Paediatrics and Adolescent Medicine, LKS Faculty of Medicine, The University of Hong Kong, Hong Kong, China; 3 Department of Pediatric, Perinatal and Maternal Medicine, Tokyo Medical and Dental University, National Defence Medical College, Tokyo, 113-8519 Japan; 4 Laboratory of Immune System Biology, National Institute of Allergy and Infectious Diseases, National Institutes of Health, Bethesda, MD 20892-1892 USA

**Keywords:** Adaptive immunity, Immunogenetics, Immunological disorders, Genetics, Immunology, Molecular biology

## Abstract

Inborn errors of immunity (IEI) are a heterogeneous group of monogenic disorders that include primary immunodeficiency’s and other disorders affecting different aspects of the immune system. Next-Generation Sequencing (NGS) is an essential tool to diagnose IEI. We report our 3-year experience in setting up facilities for NGS for diagnosis of IEI in Chandigarh, North India. We used a targeted, customized gene panel of 44 genes known to result in IEI. Variant analysis was done using Ion Reporter software. The in-house NGS has enabled us to offer genetic diagnoses to patients with IEI at minimal costs. Of 121 patients who were included pathogenic variants were identified in 77 patients. These included patients with Chronic Granulomatous Disease, Severe Combined Immune Deficiency, leukocyte adhesion defect, X-linked agammaglobulinemia, Ataxia Telangiectasia, Hyper-IgE syndrome, Wiskott Aldrich syndrome, Mendelian susceptibility to mycobacterial diseases, Hyper-IgM syndrome, autoimmune lymphoproliferative syndrome, and GATA-2 deficiency. This manuscript discusses the challenges encountered while setting up and running targeted NGS for IEI in our unit. Genetic diagnosis has helped our patients with IEI in genetic counselling, prenatal diagnosis, and accessing appropriate therapeutic options.

## Introduction

Inborn errors of immunity (IEI) are a group of phenotypically and genetically diverse disorders characterized by monogenic defects affecting human immunity^1^. Patients with IEI have an increased susceptibility to infections, autoimmunity, autoinflammation, allergy, and the development of malignancies^2,3^. Accurate diagnosis of these conditions is essential for tailoring management protocols. Population prevalence of IEI ranges from 1:1000 to 1:10,000. With the recent discovery of several novel genetic defects, the prevalence of IEI is now believed to be much higher^4,5^.

Wider usage of next-generation sequencing (NGS) platforms has resulted in increased recognition of monogenic forms of IEI in recent years. According to the 2019 International Union of Immunological Societies (IUIS) classification, 424 IEI have a genetic basis^6^.

Diagnosing IEI in the developing world is challenging due to lack of awareness, delays in clinical presentation, and limitations in the availability of necessary diagnostic techniques^7^. Variations in genotype and phenotype of IEI in different regions of the world make the diagnosis even more complex and challenging. While several monogenic defects have similar clinical phenotypes, monogenic defects in the same gene can result in varied clinical phenotypes depending on the type of the variant and its functional consequences. Molecular testing, however, is an indispensable tool for diagnosing IEI with atypical presentations^8^.

NGS has contributed significantly in terms of defining novel genes in patients where previous approaches have been unrewarding. NGS for diagnostic purposes needs high-quality sequencing data, clinically appropriate turn-around time, and affordable cost^9^.

Although NGS is routinely used in immunology laboratories the world over, commissioning, installation, and effectively running such a facility for patient care in the context of a developing country was very challenging. However, we were able to convince the hospital administration at our Institute of the urgent need to establish such a facility at the Advanced Pediatrics Centre. This required several rounds of deliberations. We herein report our preliminary experience of initiating a sequencing facility to diagnose IEI using targeted NGS.

## Materials and methods

### Participants

**Figure 1:**
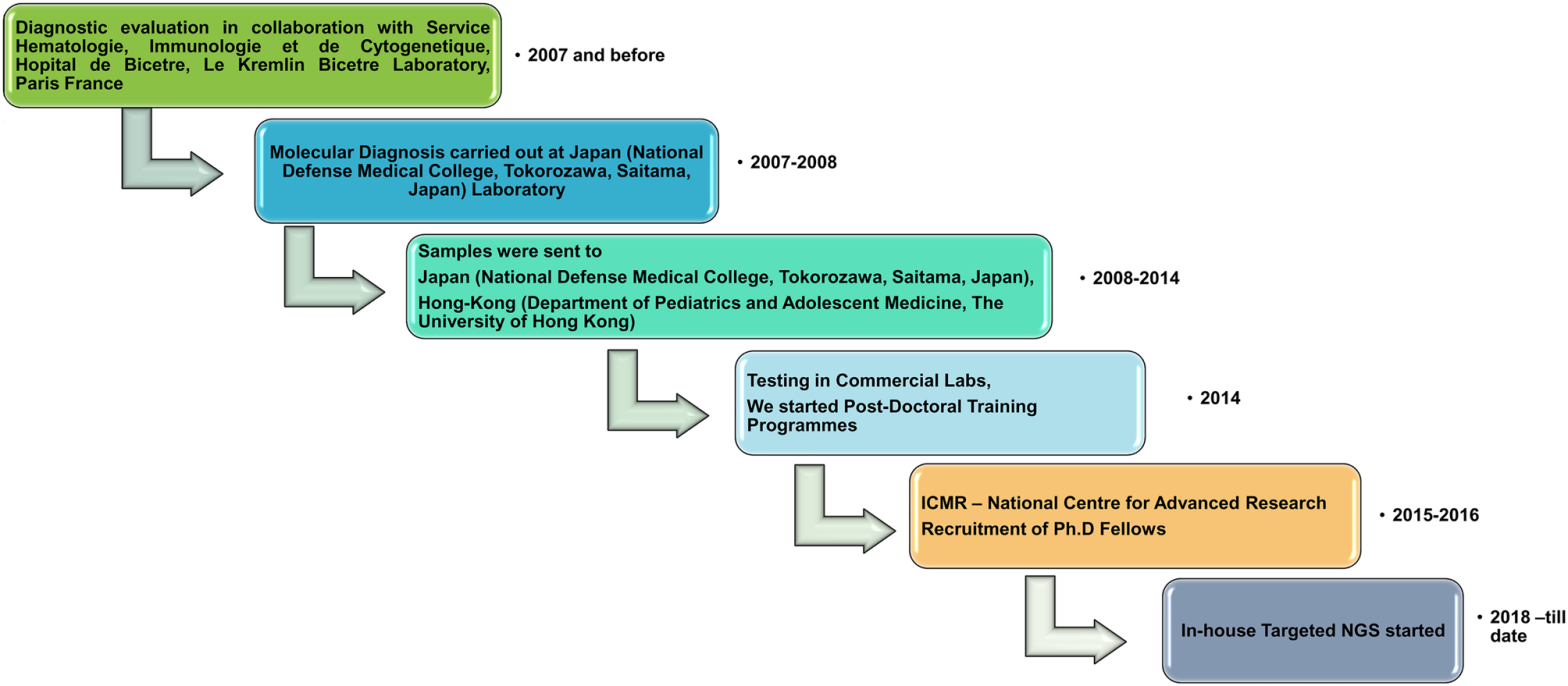
Evolution of molecular diagnosis of patients with IEIs at Allergy Immunology Laboratory, Advanced Pediatric Centre, Chandigarh, India.

Patients were enrolled in Primary Immunodeficiency Clinic, Advanced Pediatrics Centre, Postgraduate Institute of Medical Education and Research (PGIMER), Chandigarh, India after obtaining informed assent from the children and informed consent from the parents or legal guardians. All experiments were performed in accordance with guidelines and regulations outlined by the departmental review board of Advanced Pediatrics Centre, PGIMER, Chandigarh (Vide DRB-104-21). Written Informed consent was obtained from all participants and considered mandatory for participation in the study. More than 700 patients have been diagnosed to have IEI at our centre. Patients fulfilling European Society of Immunodeficiency (ESID) diagnostic criteria for various primary immunodeficiency diseases and referred or being followed up at our centre were included in the study. Patients with both possible and probable diagnosis were included cases. Molecular diagnosis was made at our centre initially with the help of collaboration with international laboratories or by sending samples to commercial laboratories. We started performing Sanger sequencing for a few common genes for IEI in 2016. We then initiated an in-house targeted NGS facility at our centre from August 2018. We have since completed targeted NGS for 121 patients with different IEI (Fig. 1).

We have included the patients in which the preliminary diagnosis of IEI was made based on clinical presentation and basic immunological investigations such as complete blood counts (CBCs), nephelometric assessment for immunoglobulins, antibody response to vaccine antigens (diphtheria and tetanus for protein antigens and pneumococcal polysaccharide for polysaccharide antigens) by enzyme-linked immunosorbent assays and lymphocyte subset analysis by flow cytometry.

### Flow-cytometry evaluation

Flow-cytometry helps in the confirmation and categorization of IEIs. Multiparametric flow cytometry helps rapidly delineate and confirm many forms of IEIs in concert with genetic diagnoses. Flow cytometry for lymphocyte subsets in patients with suspected severe combined immunodeficiency (SCID) was carried out using markers—CD45, CD3, CD19, and CD56. Btk protein expression on monocytes labelled with CD14 was assayed in patients with XLA. Diagnosis of CGD was based on the dihydrorhodamine (DHR) testing using phorbol 12-myristate 13-acetate (PMA) as a stimulant. b558 (gp91phox/p22phox), p47phox and p67phox staining was subsequently carried out to sub-categorize the type of CGD. Estimation of naïve T cells (CD45RA + CD45RO–) and memory T cells (CD45RO + CD45RA−) in CD4 + and CD8 + T lymphocyte populations was done for patients with SCID. Intracellular staining of DOCK8 in lymphocytes was used to recognize patients with DOCK8 defect. The IFN-γR1 and IL12Rβ1 assays were carried out by surface staining using anti-human CD119 and CD212 (upon stimulation with a mitogen for 72 h) respectively in patients with suspected Mendelian susceptibility to mycobacterial disease (MSMD). CD18 was estimated on neutrophils in patients with leukocyte adhesion deficiency (LAD)^10–12^.

### Targeted sequencing

Of the three major NGS strategies- (i.e. Whole Genome sequencing, Whole Exome sequencing and targeted panel gene sequencing), we chose the last option as it can be ubiquitously applied in clinical settings. Targeted NGS has the following advantages- (i) It provides disease-specific data with fewer variants of uncertain significance; hence, it simplifies bioinformatic analysis (ii) It ensures coverage and read depth for target genes of interest (iii) It obviates the need for expensive laboratory equipment and data storage facility^13^. We used the Ion Torrent S5 system from ThermoFisher Scientific for targeted NGS (details of genes in our targeted panel are listed in Table S1) in this study^14,15^.

We have recently paired the Ion S5 instrument with the Ion-Chef library preparation and chip-loading device in our setting. This has made sample processing less laborious, rapid and more accurate^16^.

#### Panel design

We selected genes in our panel based on available literature then, genetic databases for IEIs and gene defects most common in our cohort. We used Ion AmpliSeq Designer (ThermoFisher Scientific, USA) to design a 44 gene PID panel covering genes most commonly implicated in inborn errors of immunity. This custom panel comprised two pools of 672 amplicons (coverage summary of each gene in Table S1).

#### Targeted NGS library preparation and sequencing

Genomic DNA was quantified using a Qubit dsDNA HS Assay kit on QubitTM Fluorometer (ThermoFisher Scientific, USA). Five nanograms of gDNA were used for library preparation. Each sample was amplified using a custom Ion Ampliseq panel (PID 2X 2- primer pool) and HiFi mix (Thermo Fisher). PCR pools were combined for each sample and subjected to primer digestion with FuPa reagent (Thermo Fisher). The libraries were indexed; amplicons were ligated to adapters and barcodes using Ion Xpress Barcode Adapter Kit. Barcoded libraries were purified using Agencourt AMPure XP reagent (Beckman Coulter, CA) and quantified with QubitTM Fluorometer (ThermoFisher Scientific, USA). Samples were diluted and pooled for emulsion PCR.

Further, after combining the library, template preparation by emulsion PCR was done. The DNA fragment was immobilized on an Ion sphere particle (ISP) and clonally amplified. This was an automatic process performed on Ion One TouchTM 2 Instrument. This emulsion PCR results in beads with clonally amplified DNA fragments. Enrichment was done to eliminate empty beads using a robotic enrichment system (Ion One TouchTM ES). Finally, the beads containing clonal populations of DNA were obtained. Sequencing primer and polymerase were added to template positive ISPs and loaded onto Ion 530 Chip. Sequencing was done using Ion S5TM Instrument and simultaneously processed on the Ion torrent server for assembly and further analysis. The instrument was set for post-run clean-up after every run. The variant calling and analysis of results was made using Ion reporter software (ThermoFisher Scientific, USA)^17,18^. Large deletions/duplications were screened by Integrative Genomics Viewer (IGV) software using BAM files and confirmed by multiplex-ligation dependent probe amplification in selected cases.

### Sanger sequencing

Sanger validation of the identified variants in NGS was done in 20 patients. PCR products from genomic DNA were sequenced on an automated fluorescence-based sequencer (ABI 3500, Applied BiosystemsTM; Thermo Fisher Scientific, USA) using BigDyeTM Terminator (V3.1 Applied Biosystems™). Sequencing primers were the same as those used for amplification PCR. Upon sequencing, the results were obtained in .abi format and were analyzed using Codon-code aligner for DNA sequence assembly (4.2.5/2013). The patient sequence was compared with the reference human sequence obtained from the Ensemble database (https://asia.ensembl.org/index.html)^19^. Variants were classified using multiple tools including Human Gene mutation Database, ClinVar, dbSNP, and VarSome^20^. While filtering variants, all the synonymous, intronic, common variants with 1% or higher population frequency were initially excluded. Rare variants were then individually evaluated. Multiplex ligation dependent probe amplification (MLPA) was also performed to validate the large deletion/duplication as described previously^21,22^. All the variants were categorized into the following five groups- Pathogenic, Likely pathogenic, Benign, Likely benign and variant of uncertain significance (VUS)^23^. All the variants classified as benign or pathogenic in databases were considered benign or pathogenic, respectively.

### Ethics approval

The manuscript was approved by the departmental review board of the Advanced pediatrics Center, PGIMER, Chandigarh, India (Ref no. DRB-104–21).

### Consent to participate

Written informed consent was obtained from participants in the manuscript, wherever required. In case of minors the consent was obtained from the legally authorised representative.

### Consent for publication

Due consent taken for publication of clinical photographs and other clinical images. In case of minors the consent was obtained from the legally authorised representative.

## Results

Identification and evaluation of potential variants- One hundred twenty-one (121) patients (91 males, 30 female) were analysed using targeted NGS. Representative Sanger validation data have been provided in Supplementary Fig. S1-2. We could identify pathogenic variants in 63.6% (*n* = 77/121) patients. Pathogenic variants identified include *CYBB* (*n* = 13), *NCF2* (*n* = 7), *CYBA* (*n* = 1), *BTK* (*n* = 8), *RAG1* (*n* = 3), *RAG2* (*n* = 1), *ADA* (*n* = 3), *JAK3* (*n* = 2), *IL2RG* (*n* = 4), *ITGB2* (*n* = 11), *ATM* (*n* = 9), *WAS* (*n* = 2), *DOCK8* (*n* = 1), *STAT3* (*n* = 3), *IFNGR1* (*n* = 2), *IL12RB1* (*n* = 1), *CD40LG* (*n* = 2), *CD40* (*n* = 1), *GATA2* (*n* = 1), *FAS* (*n* = 2). Eight patients had biallelic variants in different PID genes (five *ATM*, one *NCF2*, *RAG1* and *JAK3* gene).

Of the thirty-three patients with suspected CGD, deleterious variants could be identified in 21 (63.6%). All had reduced or absent NADPH oxidase activity assessed by Nitroblue tetrazolium test or Dihydrorhodamine test. *CYBB* variants were present in 13, and *NCF2* in 7^24^.

We have analyzed 20 patients with suspected SCID. Lymphocyte subset was the first line of assessment for SCID patients. It delineated the immunological phenotype (T-B-NK+, T-B-NK-, T-B+ NK+) in these infants. Nearly 80% of infants died before genetic diagnosis. However, genetic counselling was done, and a prenatal diagnosis was offered for subsequent pregnancies. Pathogenic variants were detected in 13 patients- Four in *IL2RG*, three each in *RAG1* and *ADA*, two in *JAK3*, and one in *RAG2*. This was because our panel had only seven common SCID genes. *DCLER1C* gene was intentionally not included in the panel as most of the patients with Artemis defect have large deletions (involving exons 1, 2 and 3 of the *DCLER1C* gene) likely to be missed on NGS^25^.

Nine patients with suspected XLA were analyzed. NGS revealed variants in 8 patients; no variant could be detected in one patient. We identified four missense, one nonsense, one frameshift, one large deletion (See Supplementary Fig. S3) and a splice-site mutation in *BTK* in XLA patients^26^.

Twelve patients with LAD were analyzed. All had been diagnosed based on clinical presentation (omphalitis, skin and soft tissue infections, delayed umbilical cord detachment, otitis media, sepsis, skin ulcer) and CD18 expression on peripheral blood leukocytes by flow cytometry. Eleven had pathogenic variants in the *ITGB2* gene; 1 had no variant^27^. Nine patients with ataxia-telangiectasia were analyzed—all had defects in the *ATM* gene and presented with neurological defects and telangiectasia.

Five patients with WAS were analyzed—two had a defect in *WAS* gene (one stop-loss and another stop-gain variant, respectively); three had no variants. Twelve patients with suspicion of Hyper-IgE syndrome were examined for molecular defects—4 were found to have pathogenic variants (1 in *DOCK8*, 3 in *STAT3*); 7 had no variants. Laboratory investigations for patients with DOCK8 deficiency revealed eosinophilia and increased serum levels of IgE. Immunological features included low T and B cell numbers and decreased levels of serum IgM. pSTAT3 protein expression and Th17 cells were reduced in patients with STAT3 gene defects.

Nine patients with MSMD were analyzed—2 had variants in *IFNGR1*; 1 had *IL12RB1* defect; 6 had no variants in any of the genes in our targeted panel. Four patients suspected to have Hyper-IgM were analyzed- 2 had *CD40L*; 1 had *CD40* defect; 1 had no variant in any gene.

Four patients with ALPS were screened—2 had a germline *FAS* gene variant; 1 had a somatic variant in the *FAS* gene that was missed on initial analysis. The latter was detected when reanalyzed with a somatic pipeline. No variant was noted for the other two patients. A patient with Autoimmune polyendocrinopathy candidiasis ectodermal dystrophy (APECED) had an *AIRE* gene defect; this variant was not picked up by Ion reporter since there were no reads from the defined amplicon. Variant details of patients with various IEIs and corresponding flow-cytometry results have been provided in Table 1.

**Table 1:** Variant detail of patients with various IEI and corresponding flow-cytometry results.

S. No	Age	Sex	Clinical presentation	Diagnosis	*Inheritance*	Zygosity	*Gene*	*Transcript ID*	Exon	Variation	*Variant effect*	CADD Score	ACMG Classification	Flow-cytometry results
1	18 years	M	Monoarthritis	XLA	*XL*	*Hemizygous*	*BTK*	NM_000061.2	18	p.Arg618Gly	Missense	25.8	Likely Pathogenic (PM1, PM2, PP2, PP3)	Btk Protein Expression on gated CD14 + monocytes Control-83.23% SI 3.16 Patient-00.66% SI 1.03
2	11 years	M	Ompahlitis, diarrhea, Leukopenia	XLA	*XL*	*Hemizygous*	*BTK*	NM_000061.2	18	p.Gly594Glu	Missense	26.6	Likely Pathogenic (PM1, PM2, PM5, PP2, PP3)	B lymphocyte; 0.03%
3	5 years	M	Diarrhea, Bronchiectasis, pyogenic meningitis	XLA	*XL*	*Hemizygous*	*BTK*	NM_000061.2	16	p.Arg544Met	Missense	34	Pathogenic (PVS1, PM1, PM2, PM5, PP2, PP3)	Btk Protein Expression on gated CD14 + monocytes Control-60.78%SI 4.4 Patient 3.88% 2.8
4	8 months	M		XLA	*XL*	*Hemizygous*	*BTK*	NM_000061.2	2	p.Leu37Pro	Missense	26.2	Likely Pathogenic (PM1, PM2, PP2, PP3)	–
5	16 years	M	Pneumonia, Diarrhea, Measles, hepatitis, seizures	XLA	*XL*	*Hemizygous*	*BTK*	NM_000061.2	6	p.Gly173Glufs*3	Frameshift	-	Pathogenic (PVS1, PM2, PP3)	B cells : 0.02% Btk Protein Expression on gated CD14 + monocytes Control-99.45% SI 28.6 Patient-83.33%. SI 6.8
6	8 years	M	Pneumonia, Bronchiectasis	XLA	*XL*	*Hemizygous*	*BTK*	NM_000061.2	16	c.1567-2A > C	Splice-site	34	Pathogenic (PVS1, PM2, PP3)	CD3/19 + = 79.64/0.56%, BTK protein expression on monocytes = Control-98.73% SI 17.6 Patient-91.49% SI 10.8
7	11 years	M	X-linked family history (two maternal uncles death)	XLA	*XL*	*Hemizygous*	*BTK*	NM_000061.2	8	p.Arg255*	Nonsense	35	Pathogenic (PVS1, PP5, PM2, PP3)	B cells; 0.09% Btk Protein Expression on gated CD14 + monocytes Control 68.74% SI 6.8 Patient 2.37%. SI 1.8
8	1 years	M	Maternal cousin died of XLA, 2 episodes of febrile seizure	XLA	*XL*	*Hemizygous*	*BTK*	NM_000061.2		Deletion Exon 10,11	Large Deletion	–	Pathogenic	B cells: 0.47% Btk expression on CD14 + monocytes Control:98% SI 7.0 Control:91% SI 2.9
9	9 months	M	Cutaneous abscess, pneumonia, lung abscess, cervical adenitis,	CGD	*XL*	*Hemizygous*	*CYBB*	NM_000397.3	12	p.Trp516Arg	Missense	27.6	Likely Pathogenic (PM2, PM5, PP2, PP3, PP5)	Dihydrorhodamine assay (% of neutrophils showing oxidase activity) Control 93.35% SI 103 Patient 0.59%. SI 1.0
10	11 years	F	Skin abscess, pneumonia, Osteomyelitis	CGD	*AR*	*Homozygous*	*NCF2*	NM_001127651.2	9	p.Thr279Glyfs*16	Frameshift	-	Pathogenic (PVS1, PM2, PP3, PP5)	Dihydrorhodamine assay (% of neutrophils showing oxidase activity) Control 97.97%. SI 121 Patient 17.57%. SI 2.26
11	3 months	M		CGD	*AR*	*Homozygous*	*NCF2*	NM_001127651.2	9	p.Thr279Glyfs*16	Frameshift	-	Pathogenic (PVS1, PM2, PP3, PP5)	Dihydrorhodamine assay (% of neutrophils showing oxidase activity) Control 95.10%. SI 118.6 Patient 13.93%. SI. 1.3
12	1 years	M	Septicemia, pneumonia	CGD	*XL*	*Hemizygous*	*CYBB*	NM_000397.3	IVS10	c.1152-1G > A	Splice-site	34	Pathogenic (PVS1, PM2, PP3, PP5)	–
13	4 years	M		CGD	*XL*	*Hemizygous*	*CYBB*	NM_000397.3	5	p.Glu150*	Nonsense	35	Pathogenic (PVS1, PM2, PP3, PP5)	–
14	3 months	M	Pneumonia, cervical adenitis, liver abscess- multiloculated, Septicemia	CGD	*AR*	*Homozygous*	*NCF2*	NM_001127651.2	9	p.Thr279Glyfs*16	Frameshift	–	Pathogenic (PVS1, PM2, PP3, PP5)	Dihydrorhodamine assay (% of neutrophils showing oxidase activity) Control 97.26% SI 97.8 Patient 1.23%. SI 3.9
15	8 years	M	Pneumonia, submandibular adenitis	CGD	*XL*	*Hemizygous*	*CYBB*	NM_000397.3	7	p.Gly252Glufs*31	Frameshift		Pathogenic (PVS1, PM2, PP3)	Dihydrorhodamine assay (% of neutrophils showing oxidase activity) Control 89.94% SI 94.2 Patient 38.71% SI 3.2 b558 expression on Granulocyte = Control-98.21%, Patient-03.00%
16	7 months	M	Cervical lymphadenitis, multiple abscess	CGD	*XL*	*Hemizygous*	*CYBB*	NM_000397.3	12	p.Asp500Asn	Missense	27.9	Likely Pathogenic (PM2, PM5, PP2, PP3)	Dihydrorhodamine assay (% of neutrophils showing oxidase activity) Control 96.92%, SI 137.7 Patient 86.16%. SI 7.7
17	1 months	M	Fever, abdominal distension, sepsis meningitis, pneumonia	CGD	*XL*	*Hemizygous*	*CYBB*	NM_000397.3	11	p.Trp443*	Nonsense	41	Pathogenic (PVS1, PM2, PP3)	Dihydrorhodamine assay (% of neutrophils showing oxidase activity) Control 99.47% SI 230.4 Patient 12.75%. SI 1.8
18	9 months	M	Fever,PUO, Pyonephrosis, pneumonia	CGD	*AR*	*Homozygous*	*NCF2*	NM_001127651.2	9	p.Thr279Glyfs*16	Frameshift	–	Pathogenic (PVS1, PM2, PP3, PP5)	Dihydrorhodamine assay (% of neutrophils showing oxidase activity) Control 99.61% SI 272.4 Patient 37.35%. SI 2.7
19	1.5 years	M	Axillary adenitis, pneumonia, skin abscess, BCG adenitis, osteomyelitis of right foot	CGD	*AR*	*Compound Heterozygous*	*NCF2*	NM_001127651.2	13	p.His389Gln, c.1178 + 1G > A	Missense, Splice-site	22.8, 34	Benign (BA1, BP6, BP1); Pathogenic (PVS1, PM2, PP3, PP5)	Dihydrorhodamine assay (% of neutrophils showing oxidase activity) Control 97.81% SI 126.3 Patient 00.98%. SI 0.9
20	3 months	M	Abscess in right sub mandibular, pneumonia,lymphadenitis,	CGD	*XL*	*Hemizygous*	*CYBB*	NM_000397.3	1	p.Met1Arg	Missense	26.4	Pathogenic (PVS1, PM2, PP3)	Dihydrorhodamine assay (% of neutrophils showing oxidase activity) Control 95.13% SI 183.8 Patient 19.93% SI 1.5 Expression of b558 on Granulocytes = Control-66.50% Patient-11.5%
21	2 months	M	Pneumonia, lymphadenitis, abscess, ear discharge, otitis, scalp rash, GI bleed	CGD	*XL*	*Hemizygous*	*CYBB*	NM_000397.3	9	p.Val321Serfs*27	Indel	–	Pathogenic (PVS1, PM2, PP3)	Dihydrorhodamine assay (% of neutrophils showing oxidase activity) Control 87.40% SI 32.5 Patient 00.64% SI 1.0 b558 expression on Granulocyte = Control-96.36% SI 6.8 Patient-07.64%. SI 1.2
22	2 years	M	Pneumonia , lymphadenitis, neck abscess	CGD	*XL*	*Hemizygous*	*CYBB*	NM_000397.3	9	p.Ile325Phe	Missense	25.2	Likely Pathogenic (PM1, PM2, PP2, PP3, PP5)	DHR SI = Control 96.2%. SI 63.77 Patient 70.5%. SI 5.27 b558 expression on neutrophils Control 98% SI 9.0 Patient 3% SI 1.1
23	2 years	M	Abscess in left gluteal region, liver abscess	CGD	*XL*	*Hemizygous*	*CYBB*	NM_000397.3	8	p.Arg290*	Nonsense	35	Pathogenic (PVS1, PM2, PP5, PP3)	DHR = Control 93.66% Patient 0.07%
24	5 months	M	Pneumonia, fever and cough	CGD	*AR*	*Homozygous*	*NCF2*	NM_001127651.2	9	p.Thr279Glyfs*16	FrameshiftDeletion	–	Pathogenic (PVS1, PM2, PP3, PP5)	DHR = Control 99.25% SI 34 Patient 49.69%. SI 0.8 B558 expression on granulocytes Control 68% SI 6.3 Patient. 0.5% SI 1.2
25	7 years	M	Burkholderia sepsis, pneumonia, lymphadenitis, colitis	CGD	*XL*	*Hemizygous*	*CYBB*	NM_000397.3	9	p.Glu309Lys	Missense	28.1	Likely Pathogenic (PM1, PM2, PP5, PP2, PP3)	DHR SI = Control 97% SI 101.99 Patient 88%. SI. 11.44 b558 expression on granulocytes Control 99% SI 9.7 Patient 71% SI 4.5
26	8 months	M	Colitis, nasal granuloma, Septicemia, diarrhea, hypergammaglobulinemia	CGD	*XL*	*Hemizygous*	*CYBB*	NM_000397.3	6	p.Ile190_Thr191del	Nonframeshift Deletion	–	Likely Pathogenic (PM1, PM2, PM4, PP3)	DHR = Control 99.51% SI 158.7 Patient- 4.64% SI 1.9 Expression of b558 on Granulocytes = Control-97.84% SI 14.2 Patient-00.25% SI 1.2 Mother-38.68%
27	8 months	M	Ear discharge, blood in stools, pneumonia, allergic proctitis	CGD	*AR*	*Homozygous*	*CYBA*	NM_000631.4		Deletion Exon 2–4	Large Deletion	–	Pathogenic	–
28	1 year	M	Pneumonia, cervical lymphadenitis	CGD	*XL*	*Hemizygous*	*CYBB*	NM_000397.3	5	p.Arg130*	Nonsense	34	Pathogenic (PVS1, PM2, PP5, PP3)	DHR = Control 99.91% SI 161 Patient 4.65% SI 1.42 Expression of b558 on Granulocytes = Control-79.22% SI 2.7 Patient-00.2%. SI 0.7
29	4 months	M		CGD	*AR*	*Homozygous*	*NCF2*	NM_001127651.2	3	p.Arg66*	Nonsense	36	Pathogenic (PVS1, PM2, PP3, PP5)	DHR Control 85% SI 87 Patient 0.15% Si 0.98
30	2.5 months	F	Rash pneumonia, diarrhea, Purulent ear discharge, oral thrush, hepatosplenomegaly	SCID	*AR*	*Homozygous*	*RAG2*	NM_001243786.1	3	p.Trp416Leu	Missense	27.7	Likely Pathogenic (PM1, PM2, PP2, PP3)	Lymphocyte Subset (CD3/19/56/3 + 56 +)% = 7.67/0.69/82.67/0.35 CD4/CD8 ratio = Con-2.20; Pt-7.81 CD45RA + = Con-63.64%, Pt. 06.42% CD4 + CD45RA + = Con-58.06%, Pt-10.20% CD8 + CD45RA + = Con-82.31%, Pt-04.18%
31	5 months	M	Pneumonia, absent thymus, candida sepsis (blood, urine), BAL- Pseudomonas	SCID	*AR*	*Homozygous*	*RAG1*	NM_000448.2	2	p.Arg716Gln	Missense	31	Likely Pathogenic (PM1, PM2, PM5, PP2, PP3, PP5)	Lymphocyte Subset (CD3/19/56/3 + 56 +)% = 2.36/3.84/92.22/1.21%
32	1 months	F	Rash, Pneumonia, nephrotic syndrome, Failure to thrive	SCID	*AR*	*Homozygous*	*ADA*	NM_000022.2	5	p.Gly136Asp	Missense	25.7	Likely Pathogenic (PM1, PM2, PP2, PP3)	Lymphocyte Subset (CD3/19/56/3 + 56 +)% = 89.01/0.33/0.77/3.37%
33	8 months	F	Pneumonia, hepatosplenomegaly, failure to thrive, diarhhoea, Blood *Acetobacter baumanii*	SCID	*AR*	*Compound Heterozygous*	*JAK3*	NM_000215.3	8,6	p.Arg350Trp, p.Met235Thr	Missense	31, 24.2	Likely Pathogenic (PM1,PM2, PP2, PP3); Uncertain Significance (PM1, PM2, PP2)	Lymphocyte Subset (CD3/19/56/3 + 56 +)% = 11.32/69.81/1.75/14.86% CD127 Control-59.18, Patient-5.14,
34	4 years	M	Multiple episodes of pneumonia	SCID	*AR*	*Homozygous*	*ADA*	NM_000022.2	IVS6	c.478 + 6 T > A	Splice-site	24.4	Likely Pathogenic (PM2, PP3)	Lymphocyte Subset (CD3/20 +)% = 64.75/03.91% HLA DR + on CD3 T cells = Control-12.60%, Patient-91.96% CD4/CD8 ratio = Con-1.47; Pt-00.06 CD45RO + = Con-65.22%, Pt. 23.39% CD4 + CD45RO + = Con-64.15%, Pt-16.44% CD8 + CD45RO + = Con-72.60%, Pt-17.30%
35	3 years	M	Consanguinity, family history, pneumonia, failure to thrive, rash, diarrhea, absent thymus	SCID	*AR*	*Compound Heterozygous*	*RAG1*	NM_000448.2	2	p.Glu193Lys, p.Lys621Argfs*10	Missense, Frameshift	17.71, -	Benign (PP2, PP3, BS1, BS2, BP6); Pathogenic (PVS1, PM2, PP3)	Lymphocyte Subset (CD3/19/56/3 + 56 +)% = 59.82/2.25/15.06/18.94%
36	35 days	M	Pneumonia, diarrhea, failure to thrive,	SCID	*AR*	*Homozygous*	*RAG1*	NM_000448.2	2	p.Gly393Alafs*10	Frameshift	–	Pathogenic (PVS1, PM2, PP3)	–
37	8 months	M	Family history, rash, pneumonia, diarrhea, failure to thrive	SCID	*XL*	*Hemizygous*	*IL2RG*	NM_000206.2	5	p.Ser251*	Nonsense	34	Likely Pathogenic (PVS1, PM2, BP4)	Lymphocyte Subset (CD3/19/56/3 + 56 +)% = 1.37/96.34/0.90/0.50% , Expression of CD132 on L/M/N = Control-48.25/81.71/77.48, Patient-24.27/25.84/26.82%, HLA DR + T-cells = Control-15.71%, Patient-83.46%, CD4/CD8 ratio = Con-0.92; Pt-1.37 CD45RA + = Con-48.96%, Pt. 5.93% CD4 + CD45RA + = Con-42.39%, Pt-1.23% CD8 + CD45RA + = Con-56.99%, Pt-10.19%
38	9 months	M	Family history, diarrhea	SCID	*AR*	*Homozygous*	*ADA*	NM_000022.2	9	p.Arg282Leu	Missense	35	Pathogenic (PVS1, PM1, PM2, PM5, PP2, PP3)	Lymphocyte Subset (CD3/19/56/3 + 56 +)% = 53.18/1.54/24.35/13.28%
39	6 months	M	Severe pneumonia, death in neonatal period	SCID	*XL*	*Hemizygous*	*IL2RG*	NM_000206.2	2	p.Leu57His	Missense	25.5	Likely Pathogenic (PM2, PP2, PP3)	–
40	6 months	M	Fever, rash, diarrhea, pneumonia, HLH, pancytopenia, family history	SCID	*XL*	*Hemizygous*	*IL2RG*	NM_000206.2	7	c.924 + 1G > A	Splice-site	33	Pathogenic (PVS1, PM2, PP3, PP5)	Lymphocyte Subset (CD3/19/56/3 + 56 +)% = 2.46/87.07/2.51/00.64%
41	5.5 months	M	Fever, diarrhea, pneumonia, maculopapular rash, BCG site abscess, cytopenia	SCID	*AR*	*Homozygous*	*JAK3*	NM_000215.3	8	p.Arg350Trp	Missense	32	Likely Pathogenic (PM1, PM2, PP2, PP3)	Lymphocyte Subset (CD3/19/56/3 + 56 +)% = 2.52/93.25/00.41/00.57%
42	5 months	M	Rash, pneumonia, hepatosplenomegaly, BCG ulceration, failure to thrive, oral thrush	SCID	*XL*	*Hemizygous*	*IL2RG*	NM_000206.2	5	p.Glu199Valfs*76	Indel	36	Pathogenic	Lymphocyte Subset (CD3/19/56/3 + 56 +)% = 00.61/97.82/0.22/0.04% Common y chain(CD132) expression on L/M/N = Control-83.53/99.54/66.25% Patient-25.23/98.19/17.53%
43	3 months	M	Delayed separation of cord, Omphalitis, Fever, Periumblical erythema, recurrent infections, neutrophilic leukocytosis, thrombocytosis	LAD	*AR*	*Homozygous*	*ITGB2*	NM_001127491.2	14	p.Arg693*	Nonsense	43	Pathogenic (PVS1, PM2, PP3, PP5)	CD18 on Granulocyte: Control 98.56% Patient 00.18%
44	27 days	M	Swelling and redness around umblicus, Erythema around umblicus, omphalitis, necrotizing fasciitis, neutrophilic leukocytosis	LAD	*AR*	*Homozygous*	*ITGB2*	NM_001127491.2	14	p.Arg693*	Nonsense	43	Pathogenic (PVS1, PM2, PP3, PP5)	CD18 on Granulocyte : Control 98.56% Patient 00.15%
45	5 months	F	Fluid filled veicle over left thigh erythema, fever, lethargy, peeling of skin, history of loose stools, splenomegaly, sepsis, Ulcer, fever, pallor, thrombocytopenia	LAD	*AR*	*Homozygous*	*ITGB2*	NM_001127491.2	14	p.Arg693*	Nonsense	43	Pathogenic (PVS1, PM2, PP3, PP5)	CD18 on Granulocyte : Control 99.93% Patient 00.02%
46	6 years	M	Ulcer over left thigh, boil over left gluteal region, fever, single fissure present over groin hypopigmented scar, hyperlinearity of palms, edema	LAD	*AR*	*Homozygous*	*ITGB2*	NM_001127491.2		c.1224 + 4A > G	Splice-site	18.96	Likely Pathogenic (PM2, PP3)	CD18 on Granulocyte: Control 99.56% Patient 3.60%
47	4 months	F	Omphalitis, nodule like lesion in perianal area, recurrent febrile, Neutrophilic leucocytosis TLC markedly increased, Microcytic hypochromic anemia, hepatomegaly	LAD	*AR*	*Homozygous*	*ITGB2*	NM_001127491.2	7	p.Leu275Alafs*39	Frameshift	-	Pathogenic (PVS1,PM2)	CD18 on Granulocyte: Control 99.33% Patient 0.10%
48	11 days	F	Neutrophilic leukocytosis, Fever	LAD	*AR*	*Homozygous*	*ITGB2*	NM_001127491.2	12	p.Cys506Alafs*23	Frameshift	-	Pathogenic (PVS1,PM2)	CD18 on Granulocyte: Control 99.24% Patient 0.00%
49	2 months	M	Abdominal distention and persistant leukocytosis, Omphalitis, Neutrophilia	LAD	*AR*	*Homozygous*	*ITGB2*	NM_001127491.2	14	p.Arg693*	Nonsense	43	Pathogenic (PVS1, PM2, PP3, PP5)	CD18 on Granulocyte: Control 97.70% Patient 0.06%
50	10 days	M		LAD	*AR*	*Homozygous*	*ITGB2*	NM_001127491.2	7	c.897 + 1G > A	Splice-site	24.7	Pathogenic (PVS1, PM2, PP3, PP5)	CD18 on Granulocyte: Control 99.88% Patient 01.51%
51	3 months	M	Fever, loose stools, cough, Oral ulcers, Oral thrush, Umblical cord not fallen, perianal ulcer	LAD	*AR*	*Homozygous*	*ITGB2*	NM_001127491.2	13	p.Glu614*	Nonsense	37	Pathogenic (PVS1, PM2, PP3)	CD18 on Granulocyte: Control 96.10% Patient 00.11%
52	1 month	F		LAD	*AR*	*Homozygous*	*ITGB2*	NM_001127491.2	8	p.Ile316Lysfs*11	Frameshift	–	Pathogenic (PVS1, PM2, PP3)	CD18 on Granulocyte: Control 99.89% Patient 00.27%
53	1 month	M		LAD	*AR*	*Homozygous*	*ITGB2*	NM_001127491.2	14	c.1878-1G > A	Splice-site	24	Pathogenic (PVS1, PM2, PP3)	CD18 on Granulocyte: Control 99.43% Patient 00.96%
54	7 years	F	Oculomotor apraxia	Ataxia telangiectasia	*AR*	*Homozygous*	*ATM*	NM_000051.3	46	p.Gln2220*	Nonsense	40	Pathogenic (PVS1, PP5, PM2, PP3)	AFP- 178
55	3 years	F	Oculomotor apraxia	Ataxia telangiectasia	*AR*	*Homozygous*	*ATM*	NM_000051.3	2	p.Arg23*	Nonsense	36	Pathogenic (PVS1, PP5, PM2, PP3)	AFP- 52.68
56	9 years	M	Oculomotor apraxia, Neuroregression,	Ataxia telangiectasia	*AR*	*Compound Heterozygous*	*ATM*	NM_000051.3	24, 50	p.Asn1183Trpfs*16, p.Arg2486*	Frmashift Deletion, Nonsense	39	Pathogenic (PVS1, PP5, PM2, PP3)	AFP- 286.3
57	2 years	F	Oculomotor apraxia, Ataxia, telangiectasia	Ataxia telangiectasia	*AR*	*Homozygous*	*ATM*	NM_000051.3	2	p.Arg23*	Nonsense	36	Pathogenic (PVS1, PP5, PM2, PP3)	AFP- 309
58	8 years	F	Oculomotor apraxia, ocular telangiectasia	Ataxia telangiectasia	*AR*	*Homozygous*	*ATM*	NM_000051.3	42	p.Arg2034*	Nonsense	37	Pathogenic (PVS1, PP5, PM2, PP3)	AFP- 611.9
59	6 years	F	Oculomotor apraxia, Tonsilitis	Ataxia telangiectasia	*AR*	*Compound Heterozygous*	*ATM*	NM_000051.3	2, 20	p.Arg23*, c.3077 + 1G > T	Nonsense, Splice-site	36, 35	Pathogenic (PVS1, PM2, PP3, PP5)	AFP- 123.38
60	9 years	F	Cerebral atrophy	Ataxia telangiectasia	*AR*	*Compound Heterozygous*	*ATM*	NM_000051.3	3	p.Arg35*, c.497-6 T > TC	Nonsense, Splice-site	34	Pathogenic (PVS1, PP5, PM2, PP3); Uncertain Significance (PM2, BP4)	AFP- 538
61	2 years	M	Oculomotor apraxia	Ataxia telangiectasia	*AR*	*Compound Heterozygous*	*ATM*	NM_000051.3	37,49	p.Phe1877Leufs*39 p.Arg2436Lys	Framshift,Missense	33	Uncertain Significance (PM2, PP2, BP4); Pathogenic (PVS1, PM1, PM2, PP2, PP3)	AFP- 146
62	10 years	M	Gut abnormality, Ataxia, ocular telangiectasia, cerebellar atrophy, recurrent sinopulmonary infections	Ataxia telangiectasia	*AR*	*Compound Heterozygous*	*ATM*	NM_000051.3	37, 49	p.Phe1877Leufs*39 p.Arg2436Lys	Framshift,Missense	33	Uncertain Significance (PM2, PP2, BP4); Pathogenic (PVS1, PM1, PM2, PP2, PP3)	AFP- 566
63	1 year	M	Pneumonia, diarrhea, eczema, skin bleed, family history	WAS	*XL*	*Hemizygous*	*WAS*	NM_000377.2	12	p.*503Argext*79	Stop-loss	20.5	Likely Pathogenic (PM2, PM4)	WAS Protein expression: Control 99.77% Patient 99.88%
64	1 year 2 months	M		WAS	*XL*	*Hemizygous*	*WAS*	NM_000377.2		p.Arg321*	Nonsense	33	Pathogenic (PVS1, PM2, PP5, PP3)	–
65	11 years	M		AR Hyper IgE	*AR*	*Homozygous*	*DOCK8 deficiency*	NM_203447.3	23	p.Ser948*	Nonsense	38	Pathogenic (PVS1, PM2, PP3)	–
66	3 years	M	Recurrent pneumonia, skin infections, eczema, coarse facis	Hyper IgE	*AD*	*Heterozygous*	*STAT3*	NM_139276.2	16	p.Ile467Phe	Missense	28.6	Likely Pathogenic (PM2, PP2, PP3)	STAT3 Expression: Control-59%, Patient-53.7% Th17 Expression: Control- 0.6%, Patient-0.2%
67	8 years	M	Chronic eczema, recurrent cold abscess, NIH score 31,	Hyper IgE	*AD*	*Heterozygous*	*STAT3*	NM_139276.2	14	p.Arg423Gln	Missense	32	Likely Pathogenic (PM2, PP5, PP2, PP3)	STAT3 expression was reduced in patient
68	9 years	F	Coarse facial features, crowding of teeth, multiple soft tissue abscess, pneumonia, meningitis	HIGE	*AD*	*Heterozygous*	*STAT3*	NM_139276.2	22	p.Phe710Ser	Missense	32	Likely Pathogenic (PM1, PM2, PP2, PP3)	Reduced Th17 cells in patient
69	6 years	M	Multifocal non-tubercular mycobacterial osteitis	MSMD	*AD*	*Heterozygous*	*IFNGR1*	NM_000416.2	6	p.Asn274Hisfs*2	Frameshift	-	Pathogenic (PVS1, PP5, PM2)	CD119 Expression = Control-100% Patient-97.7%
70	5 years	F	Disseminated Tuberculosis with multifocal osteomyelitis	MSMD	*AR*	*Homozygous*	*IFNGR1*	NM_000416.2	1	p.Met1Ile	Missense	24.1	Pathogenic (PVS1, PM2, PP3)	Expression of CD119(IFN-γR1) on Granulocyte/Monocyte/Lymphocyte: Control-99.40,98.86,89.38% Patient-44.78,06.97,08.71%
71	8 months	F	Abdominal distension, fever, non healing left axillary ulcer, multiple swelling of axillary, neck and B/L inguinal region, Pallor, Supparative lymphadenitis	MSMD	*AR*	*Homozygous*	*IL12RB1*	NM_001290024.1	14	c.1738 + 2T > A	Splice-site	33	Pathogenic (PVS1, PM2, PP3)	Expression of CD212 (IL12R-β1) = Control-48.09%, Patient-7.45%
72	8 years	M	Fever, diarrhea, multiple infections, oral thrush	GATA2	*AD*	*Heterozygous*	*GATA2*	NM_032638.4	2	p.Arg67Serfs*10	Frameshift	-	Pathogenic (PVS1, PM2, PP3)	Reduced B-cells
73	6 years	M		ALPS	*AD*	*Heterozygous*	*FAS*	NM_000043.4	3	p.Gly66Asp	Missense	25.6	Likely Pathogenic (PP3, PM1, PM2, PP2)	Double negative T lymphocyte = 2.46%
74	9 years	M	Pallor, hepatosplenomegaly, pancytopenia	ALPS	*AD*	*Heterozygous*	*FAS*	NM_000043.4	9	p.Arg250Gln	Missense	26.5	Likely Pathogenic (PM1, PM2, PM5, PP5, PP2, PP3)	Double negative T lymphocyte = 2.59%
75	11 years	M		Hyper IgM	*XL*	*Hemizygous*	*CD40LG*	NM_000074.2	5	p.Tyr169_Ile171del	Noframeshift Deletion	–	Likely Pathogenic (PM1, PM2, PM4, PP3)	Expression of CD40L on activated CD69/4 + T cell: Control-95.85% Patient-1.87%
76	40 years	F		Hyper IgM	*XL*	*Hemizygous*	*CD40LG*	NM_000074.2	1	p.Lys52Lys	Synonymous	23.1	Likely Pathogenic (PVS1, PM2, PP3)	–
77	4 years	F	Eosinophilia, recurrent infections, pneumonia, skin abscess, diarrhea, stool- Giardia	Hyper IgM	*XL*	*Homozygous*	*CD40*	NM_001250.5	4	p.Cys103*	Nonsense	32	Pathogenic (PVS1, PM2, PP3)	Class switch defect Increased B-cell: Control-52.27% Patient-88.24% Decreased switched B-cells: Control-30.68% Patient-0.39%

## Discussion

Advances in genetic technology have rapidly changed healthcare delivery in low- and middle-income countries. NGS utilization has decreased the time to diagnosis, increased the diagnostic rate, and provided valuable insight into the genotype–phenotype correlation of IEI in a timely and cost-effective way^28,29^. IEI is not uncommon in India; however, their diagnosis is either missed or delayed due to a lack of awareness and a paucity of diagnostic facilities. There is an urgent need to increase testing capacity for early recognition, diagnosis, and management of IEI in our country^30–32^.

We have been diagnosing patients with IEI at our centre for the past 25 years. However, services for molecular diagnosis for IEI both in government and commercial sectors have not been available in India until 2016. For molecular diagnosis of IEI, we established academic collaboration with Service Hématologie, Immunologie et de Cytogénétique, Hôpital de Bicêtre, Le Kremlin Bicêtre, at France in the year 2007. Later, we established collaboration with institutes at Japan (National Defense Medical College, Saitama) and Hong Kong (Department of Paediatric and Adolescent Medicine, University of Hong Kong) in the years 2008 and 2010, respectively. This has facilitated molecular diagnosis for many of our patients with IEI. Our centre was designated as Centre for Advanced Research in diagnosis and treatment for primary immunodeficiency diseases by the Indian Council of Medical Research, Government of India, in 2015. Until 2016, tests available for diagnosis of IEI at our centre include immunoglobulin estimation, NBT, and flow cytometry for several surface and intracellular proteins^10^. With the increase in patients diagnosed with IEI in the last few years, we felt the need to establish molecular analysis at our centre^4^. We initiated Sanger sequencing for *BTK*, *CYBB*, and *WAS* genes in our centre in 2016 (Fig. 1).

Commercial laboratories in India came up with facilities (targeted exome) for molecular diagnosis of IEI in 2016. Costs incurred for sequencing in commercial laboratories were exorbitant (USD 400–500) in 2016 that later reduced in the subsequent years (USD 200 currently). The introduction of targeted NGS for IEI in 2018 at our centre has enabled us to offer this diagnostic modality to many of our patients who could not afford the costs of commercial testing. We have also been able to diagnose more IEIs each year and at a much faster pace than in previous years. The cost of targeted genetic sequencing at our setup is USD 83 per sample. This is much less than the costs incurred at commercial laboratories in India^33^. In addition, infants less than one year are covered under the JSSK (Janani Sishu Suraksha Karyakram) scheme of the Government of India. They are entitled to avail of NGS free of cost. Our Institute also provides free diagnostic services to patients from low-income groups who cannot afford the NGS charges, and charges are minimal for those who can afford this facility.

We have worked upon and improvised the standard protocol of NGS to suit our setup. We made some ingenious modifications to the recommended protocol to reduce the cost per sample and accommodate more patient samples in each run. Towards this end, we have successfully used half the recommended volume of reagents (however, concentration remained the same) at each successive step by starting with an initial DNA volume of 2.5µL instead of 5µL. So, a larger number of patient samples could be accommodated in each run. We have effectively run 42 patient samples with a 24-reaction reagent kit for 24 samples.

NGS sample preparation is a tedious and labour-intensive process requiring focus and concentration at each successive step^34,35^. After chip-loading and sequencing, we did not get results for two runs. On both these occasions, instead of repeating from the start, we started after the library quantification step as we were sure about the quality of the library preparation. So, restarting with the template preparation step instead of beginning from the start in the case of a failed run could be a helpful strategy if we are sure about the quality of library preparation.

We describe preliminary results of targeted NGS in 121 patients with different forms of IEIs diagnosed and managed at our centre. Our variant pick-up rate of 63.6% is much higher than previous studies- 25% by Yska et al. in 2019 and 29% by Vorsteveld et al. in 2021^28,36^. The pick-up rate of variants in other studies were 16%^7^ (Gallo et al., Italy, 2016), 14% (Kojima et al., Japan, 2016)^37^, 2.1% (Sun et al., China in a cohort of infants)^38^, 28.6% (Cifaldi et al., Italy, 2020)^18^ and 42.4% (Arunachalam et al., India, 2020)^33^.

There are several reasons for a higher diagnostic yield in our study. Careful patient selection with a high pre-test probability based on clinical manifestations and preliminary immunological investigations was done. Patients with a high likelihood of having a pathogenic variant in one of 44 genes included in the gene panel are sorted out in consultation with clinicians trained in immunology and have broad experience in caring and managing patients with IEI. Currently more than 400 genes are implicated in various IEI. However, we selected 44 genes based on the most common diseases we encounter at our centre and also since we aimed to provide genetic diagnosis to maximum number of patients at an affordable cost. A large panel although more desirable would be costlier to design and in addition fewer samples would be accommodated in each run. Samples of patients who are very likely to have genetic variants in the genes included in the panel were included based on clinical history and initial immunological investigations. Patients with IEI not clearly delineated upon initial immunological investigations are referred for a clinical exome or whole-exome analysis. This analysis is outsourced to commercial laboratories providing these services at an affordable cost.

NGS has facilitated the early diagnosis of patients with IEI in situations where flow cytometry was either not conclusive or did not match the clinical presentation. For instance, patient 56 was clinically suspected of having an autosomal recessive hyper-IgM was found to have biallelic variants in the *ATM* gene. Hence, relying solely on typical manifestations of the IEI may not be ideal, and a rapid genetic diagnosis is indispensable^39^.

**Figure 2:**
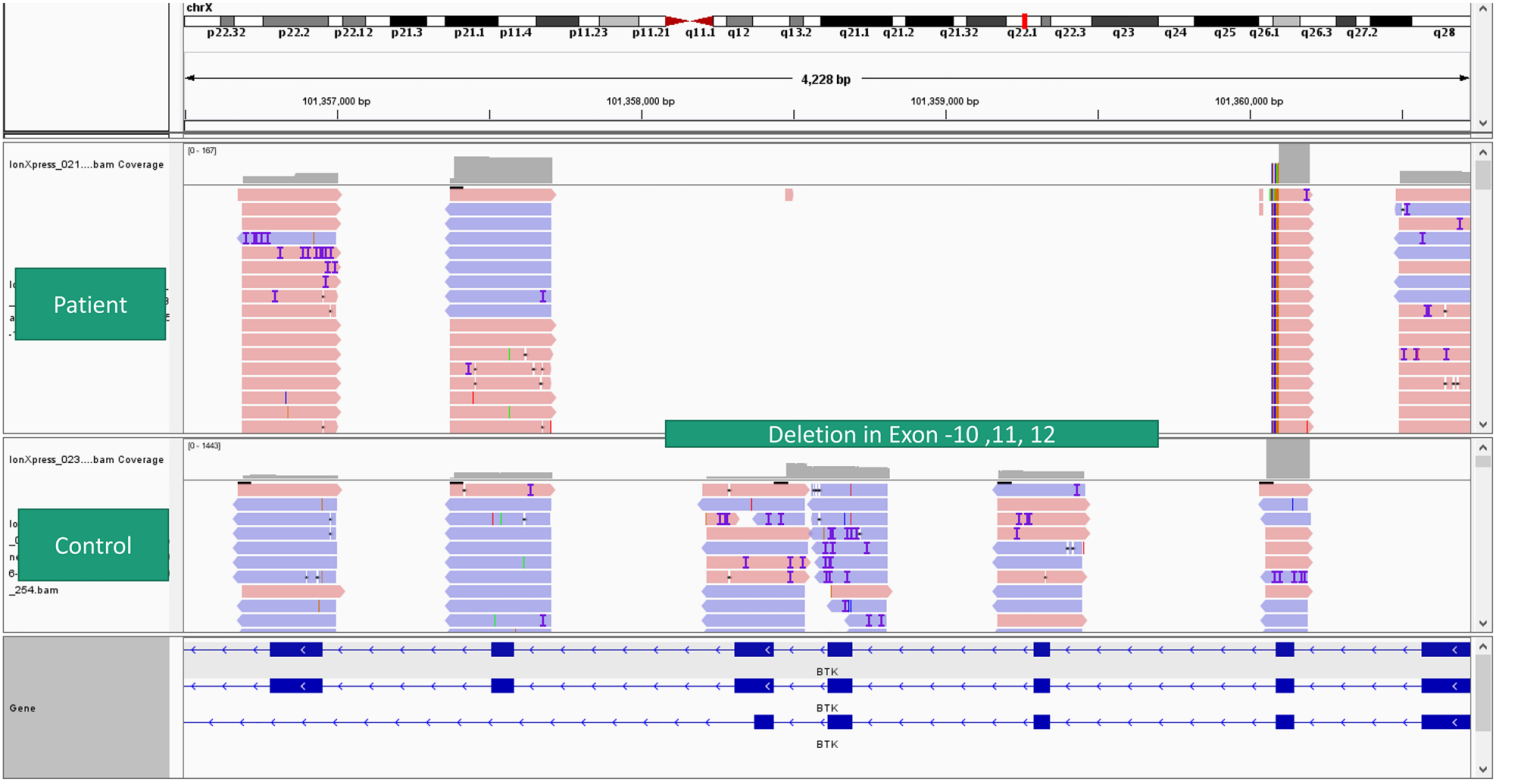
Large deletion of Exon- 10 to 12 in *BTK* gene on Integrative Genome Viewer.

There have also been instances when the initial analysis on the Ion Reporter did not reveal a pathogenic variant. In patient 8 with clinically suspected XLA, no pathogenic variant was detected at initial analysis. There was a strong clinical suspicion of XLA in this case; we manually visualized the data on Integrative Genomics Viewer (IGV). We found a large deletion of exon-10, 11 and 12 in the *BTK* gene (Fig. 2)^40^. Similarly, in another patient with suspected CGD (Pt.27), a large deletion was found in the *CYBA* gene, which was missed by the ion reporter software but was detected on manual reanalysis and visualization on the IGV. Patient 42 had an indel in *IL2RG* gene. In patient 42, analysis by the Ion reporter software revealed two *IL2RG* variants in close proximity, which was confusing. However, upon visualization of the BAM file on IGV, we realized that it was an indel (insertion of 3 nucleotides and deletion of 8 nucleotides) which was misinterpreted as two variants by the ion Reporter software.

Hence, manual data visualization on IGV and manual analysis of annotated vcf files instead of relying on variants detected by initial analysis by software is crucial. We have been able to detect these variants in these cases using this strategy.

**Figure 3:**
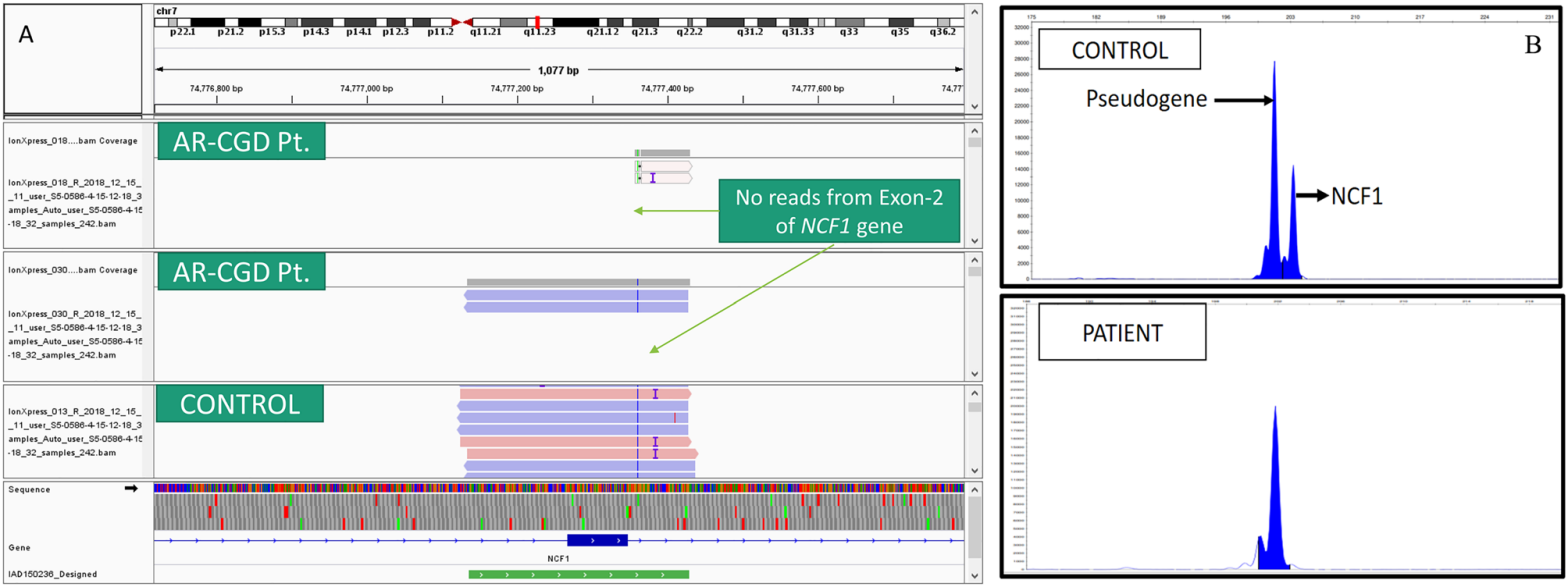
(**A**) IGV snapshot showing no reads from Exon-2 of *NCF1* gene in 2 patients with AR-CGD (**B**) Gene Scan for Exon 2 *NCF1* gene from control and an AR-CGD patient with no reads from exon 2 of *NCF*1 gene.

Detection of genetic variants in genes with known pseudogene is another problem that we encountered in our patient cohort. We faced this difficulty in patients with autosomal recessive CGD due to *NCF1* gene defect. The targeted NGS panel systematically missed the most common pathogenic variant in *NCF1*, i.e., deletion of two nucleotides at the start of Exon-2. *NCF1* gene has two flanking pseudogenes (ΨNCF1)^41^. We assume that the amplicon designed for exon-2 of the *NCF1* gene was unable to bind to its target, and thus, there was no amplification of this region, resulting in no reads for exon-2 in these patients. We performed a gene scan in 3 patients who had no reads in Exon-2 of the *NCF1* gene to check for this variant and confirmed *NCF1* GT deletion in all 3 of these patients (Fig. 3A,B).

We have also been able to offer prenatal services to many patients. Patient 40 was clinically suspected of having SCID but had expired before a genetic defect could be established. His mother was pregnant at this time, and the period of gestation was 13 weeks. We were able to identify a splice-site variant in the *IL2RG* gene in this family with X-linked SCID, and the mother was offered prenatal diagnosis by chorionic villous sampling. Molecular confirmation of diagnosis helped the family to get timely antenatal testing and appropriate genetic counselling. For some patients, especially SCID, rapid diagnosis through targeted NGS has saved lives, or genetic counselling has prevented an affected child in the subsequent pregnancy.

Pt 76 was the mother of a deceased child suspected to have X-linked Hyper-IgM, but a genetic diagnosis could not be established during the child’s life. Targeted NGS revealed a synonymous variant in exon 1 of the *CD40LG* gene proximal to donor splice-site. In-silico prediction for this variant was found to be ‘damaging’ by Mutation Taster2. Synonymous variants involving canonical splice-sites can also be pathogenic and should not be filtered out.

Genetic findings were beneficial in providing genetic counselling to affected families, carrier screening, and prenatal diagnosis. Moreover, genetic information is required for devising appropriate transplantation related strategies. Genetic findings were also crucial in deciding the treatment modalities in a few cases. Cases harbouring defects leading to antibody deficiencies were placed on regular replacement intravenous immunoglobulin therapy.

## Limitations

Some apparent limitations are intrinsic to these types of studies. The list of genes involved in the pathogenesis of immune-related diseases is continuously increasing at an exponential rate, so some of the recently discovered genes (e.g., *RIPK1, ICOSLG*, and *CYBC1*) were not included in our NGS panel. Copy number variations (CNVs), as NGS adaptation to CNV testing requires additional bioinformatics and analytical efforts. It is pertinent to mention that CNVs seem to be uncommon for PID patients. However, CNV changes were very well described for *IL7R* and *DOCK8* genes^42,43^.

We have also missed few variants with low coverage or absence of reads in that particular amplicon. Semiconductor-based sequencing are also fraught with inaccuracies in sequencing genomic regions with homopolymer repeats of the same nucleotide. This stems from an erroneous measurement of the magnitude of the voltage pulse in stretches of homopolymer repeats in the genome^44^.

Heterozygous exonic deletions could not be detected reliably using an amplicon sequencing approach. Large deletions are also not detected by NGS and may be missed unless BAM files are visually inspected on Integrative Genomics Viewer.

Another limitation of the present study is that not all genetic variants detected by NGS were validated by Sanger sequencing. While analyzing the data, we have to be cautious as no reads in an exon can be confused with deletions. In some patients, we were not able to detect any pathogenic variants. This may be due to the presence of defects in genes that are not included in our panel.

## Conclusion

The attainment of NGS use would require an amalgamation of knowledge based on clinical, immunological and molecular data and association among diverse experts in these fields. A clear description of clinical phenotype and immunological test results for NGS-based diagnostics is essential for several disease-specific features. The possibility of performing pedigree analysis and immunological follow-up is an important step relevant to understanding a given patient’s disease manifestations^45^.

A better clinical, immunological and genetic description of new IEI will meaningfully contribute to identifying diagnostic and prognostic markers and early individual therapeutic strategies with significant benefits for patients. In summary, this study describes our nascent experience in using NGS as a tool for the genetic diagnosis of IEI and discusses the expected and unexpected findings obtained. The cases described illustrate the heterogeneity and complexity encountered by professionals involved in the clinical management and genetic diagnosis of these disorders. We have also highlighted the difficulties encountered in setting up and running this facility in the context of a developing country.

## Supplementary Information

Supplementary Information.
