# Supplementary material for: Utility of targeted next generation sequencing for inborn errors of immunity at a tertiary care centre in North India
Source: Sci Rep. 2022 Jun 21;12:10416. doi: 10.1038/s41598-022-14522-1 (PMC9213413; doi:10.1038/s41598-022-14522-1)
Supplement: Supplementary file 1 — Supplementary Information. [file 41598_2022_14522_MOESM1_ESM.docx]

**Utility of targeted next generation sequencing for inborn errors of immunity at a tertiary care centre in North India**

Amit Rawat^1,*, #^, Madhubala Sharma^1,#^, Pandiarajan Vignesh^1^_,_ Ankur Kumar Jindal^1^, Deepti Suri^1^, Jhumki Das^1^, Vibhu Joshi^1^, Rahul Tyagi^1^, Jyoti Sharma^1^, Gurjit Kaur^1^, Yu-Lung Lau^2^ , Kohsuke Imai^3^, Shigeaki Nonoyama^3^, Michael Lenardo^4^, Surjit Singh^1^

**Table S1- Listing 44 gene of our Targeted PID panel and coverage summary of each gene.**

| **S. No.** | **Name** | **Chromosome**  **Number** | **Number of Amplicons** | **Total Bases** | **Covered Bases** | **Missed Bases** | **Overall Coverage** | **Number of Exons** |
| --- | --- | --- | --- | --- | --- | --- | --- | --- |
| 1 | *C1QA* | 1 | 4 | 758 | 758 | 0 | 1 | 2 |
| 2 | *C1QB* | 1 | 4 | 782 | 782 | 0 | 1 | 2 |
| 3 | *C1QC* | 1 | 5 | 758 | 758 | 0 | 1 | 2 |
| 4 | *CFH* | 1 | 38 | 3940 | 3940 | 0 | 1 | 23 |
| 5 | *FASLG* | 1 | 6 | 886 | 886 | 0 | 1 | 5 |
| 6 | *NCF2* | 1 | 15 | 1731 | 1731 | 0 | 1 | 15 |
| 7 | *FAS* | 10 | 10 | 1098 | 1098 | 0 | 1 | 10 |
| 8 | *PRF1* | 10 | 9 | 1688 | 1688 | 0 | 1 | 2 |
| 9 | *ATM* | 11 | 70 | 9791 | 9791 | 0 | 1 | 62 |
| 10 | *IL10RA* | 11 | 10 | 1807 | 1807 | 0 | 1 | 7 |
| 11 | *RAG1* | 11 | 11 | 3142 | 3142 | 0 | 1 | 1 |
| 12 | *RAG2* | 11 | 6 | 1594 | 1594 | 0 | 1 | 1 |
| 13 | *AICDA* | 12 | 5 | 647 | 647 | 0 | 1 | 5 |
| 14 | *LIG4* | 13 | 10 | 2746 | 2746 | 0 | 1 | 1 |
| 15 | *CYBA* | 16 | 7 | 648 | 648 | 0 | 1 | 6 |
| 16 | *STAT3* | 17 | 22 | 2543 | 2503 | 40 | 0.984 | 25 |
| 17 | *IL12RB1* | 19 | 18 | 2431 | 2296 | 135 | 0.944 | 22 |
| 18 | *JAK3* | 19 | 25 | 3605 | 3437 | 168 | 0.953 | 23 |
| 19 | *CTLA4* | 2 | 5 | 712 | 712 | 0 | 1 | 5 |
| 20 | *ICOS* | 2 | 6 | 650 | 650 | 0 | 1 | 5 |
| 21 | *STAT1* | 2 | 24 | 2487 | 2487 | 0 | 1 | 24 |
| 22 | *ADA* | 20 | 12 | 1212 | 1212 | 0 | 1 | 12 |
| 23 | *CD40* | 20 | 11 | 964 | 952 | 12 | 0.988 | 11 |
| 24 | *AIRE* | 21 | 18 | 1778 | 1677 | 101 | 0.943 | 14 |
| 25 | *IFNGR2* | 21 | 7 | 1084 | 1001 | 83 | 0.923 | 7 |
| 26 | *IL10RB* | 21 | 7 | 1048 | 1048 | 0 | 1 | 7 |
| 27 | *ITGB2* | 21 | 24 | 2460 | 2450 | 10 | 0.996 | 16 |
| 28 | *IL17RA* | 22 | 18 | 2731 | 2526 | 205 | 0.925 | 13 |
| 29 | *NCF4* | 22 | 11 | 1355 | 1355 | 0 | 1 | 11 |
| 30 | *GATA2* | 3 | 8 | 1493 | 1493 | 0 | 1 | 6 |
| 31 | *LRBA* | 4 | 76 | 9162 | 9162 | 0 | 1 | 58 |
| 32 | *IL7R* | 5 | 9 | 1460 | 1460 | 0 | 1 | 8 |
| 33 | *IFNGR1* | 6 | 9 | 1540 | 1540 | 0 | 1 | 7 |
| 34 | *IL17F* | 6 | 4 | 522 | 522 | 0 | 1 | 3 |
| 35 | *STX11* | 6 | 4 | 874 | 874 | 0 | 1 | 1 |
| 36 | *NCF1* | 7 | 14 | 1283 | 1271 | 12 | 0.991 | 11 |
| 37 | *DOCK8* | 9 | 50 | 6780 | 6780 | 0 | 1 | 49 |
| 38 | *BTK* | X | 19 | 2272 | 2272 | 0 | 1 | 21 |
| 39 | *CD40LG* | X | 6 | 836 | 836 | 0 | 1 | 5 |
| 40 | *CFP* | X | 10 | 1500 | 1500 | 0 | 1 | 9 |
| 41 | *CYBB* | X | 13 | 1843 | 1843 | 0 | 1 | 13 |
| 42 | *FOXP3* | X | 12 | 1406 | 1406 | 0 | 1 | 11 |
| 43 | *IL2RG* | X | 8 | 1190 | 1190 | 0 | 1 | 8 |
| 44 | *WAS* | X | 12 | 1629 | 1604 | 25 | 0.985 | 12 |

**Supplementary Fig. S1**

**Supplementary Fig. S1:** Representative image showing Sanger sequencing based validation of pathogenic variants in comparison to control (A) Insertion of single adenine nucleotide in P21 (c.960_961insA; p.Val321fs) in *CYBB* gene leading to CGD condition (B) Missense variant in *BTK gene* (c.1853A>G; p.R618G) in P1, leading to XLA (C) Patient 43 with LAD had a nonsense variant in *ITGB2 gene* (c.2077C>T; p.Arg693Ter) (D) Revealing a frameshift variant in *NCF-2* gene (c.835_836delAC p.Thr279fs) leading to CGD.

**Supplementary Fig. S2**

**Supplementary Fig. S2:** Representative images of (A) IGV based detection of INDEL, and (B) Sanger sequencing validation of indel (c.596_598delinsTGGATTATAAT) in the patient P42 (B-2) showing Insertion of TGGATTAT (highlighted) and deletion of AAC in the *IL2RG leading to* E199Vfs*77 frameshift termination in comparison to the wild type sequence in control (B-1).

**Supplementary Fig. S3**

**Supplementary Fig. S3**: Representative image showing multiplex ligation dependent probe amplification (MLPA) data revealing copy number variations in XLA case P8. A-B) Electropherogram and ratio chart of the mother revealing deletion of exon 10-11 in one allele indicating carrier status. C-D) Electropherogram and ratio chart of the case revealing deletion of exon 10-11.
